# The TLR3 Agonist Poly Inosinic:Cytidylic Acid Significantly Augments the Therapeutic Activity of an Anti-CD7 Immunotoxin for Human T-Cell Leukaemia

**DOI:** 10.3390/biomedicines7010013

**Published:** 2019-02-16

**Authors:** David J. Flavell, Suzanne E. Holmes, Sarah L Warnes, Sopsamorn U. Flavell

**Affiliations:** The Simon Flavell Leukaemia Research Laboratory, Southampton General Hospital, Southampton SO16 6YD, UK; suzanneh@leukaemiabusters.org.uk (S.E.H.); sarahwarnes@hotmail.co.uk (S.L.W.); beef@leukaemiabusters.org.uk (S.U.F.)

**Keywords:** immunotoxin, CD7, T-ALL, SCID mouse, TLR3 agonist, therapy, augmentation

## Abstract

We have previously shown that antibody-dependent cellular cytotoxicity (ADCC) cooperates with immunotoxin (IT)-mediated killing of human leukaemia cells in an severe combined immunodeficient (SCID) mouse model of human T-cell acute lymphoblastic leukaemia (SCID-HSB-2 mice), but not in an equivalent non-obese diabetic (NOD)/SCID mouse model. In these earlier studies, we reasoned that diminished ADCC due to the functional deficit in natural killer (NK) cell activity in NOD/SCID mice resulted in a failure of effective perforin/granzyme-mediated cytotoxicity necessary for the delivery of the augmentative effect. Poly-inosinic-cytidylic acid [poly (I:C)] is a synthetic dsRNA toll-like receptor 3 (TLR3) agonist that possesses a number of biological properties that includes the in vivo activation of NK cells. We show here that intravenous (i.v.) injection of SCID mice with [poly (I:C)] results in characteristic time-related changes in serum interleukin 2 (IL-2), IL-12, and interferon γ (INFγ) cytokine levels that are consistent with TLR3 driven activation of SCID mouse NK cells. Concomitantly, there are changes in the expression levels of CD2, CD16/32 (FcγRII/RIII), CD161 (NK1.1), and F4/80 in the bulk splenocyte population. These observed changes correlate with an increase in the in vitro lytic capabilities of putative NK cells from within the splenocyte population of [poly (I:C)] treated SCID mice. We demonstrate that the in vivo activation of NK cells with [poly (I:C)] in SCID mice bearing disseminated human T-cell leukaemia xenografts resulted in a significant improvement in the therapeutic activity exerted by an intact murine monoclonal antibody against human CD7. This was also seen for a saporin-based immunotoxin constructed with the same intact antibody (HB2-SAPORIN), but not with an F(ab’)_2_ derivative of the same antibody or of an IT constructed with the same F(ab’)_2_ HB2 antibody derivative. This study further demonstrates the previously reported reinforcing role of ADCC for the therapeutic activity of IT in an SCID mouse model of human T-ALL and the potential to significantly boost this further with [poly (I:C)]. Our study provides the rationale to justify the exploration of the clinical utility of IT based therapeutics in combination with TLR3 agonists, such as [poly (I:C)], for the treatment of haematological, and possibly other, malignancies.

## 1. Introduction

In pre-clinical studies, the evaluation of the in vivo therapeutic efficacy of immunotoxins (IT) and other antibody-drug conjugates (ADC) is often made in immunodeprived mouse xenograft models of human cancer [1]. Previous studies from this laboratory in a severe combined immunodeficient (SCID) mouse model of human T-cell acute lymphoblastic leukaemia (T-ALL) have shown that when the IT is constructed with an intact murine antibody, the Fc domain of the antibody is capable of recruiting host cytotoxic effector cells via antibody-dependent cellular cytotoxicity (ADCC) that acts co-operatively with the ribosome inactivating protein domain of the IT molecule to kill target leukaemia cells in vivo significantly more effectively [2].We were able to demonstrate that the systemic depletion of SCID mice of their natural killer cells (NK cells) with anti-asialo GM1 antibody [3] resulted in a significant reduction in the in vivo therapeutic activity of the anti-CD7 IT HB2-SAPORIN for CD7^+^ target HSB-2 cells. At the time, this was taken to indicate that NK cells were the major, if not the sole, cytotoxic effector cell engaging in ADCC in this model system. More recently, experimental findings suggest that the poly immunoglobulin (pIg) anti-Asialo GM1 antibody that was used in our original study also depletes macrophages. This serves to confound our original view that NK cells are the sole cytotoxic effector population in our SCID-HSB-2 model [4].

In addition to demonstrating that the therapeutic reinforcement effect due to ADCC is immunospecific for IT target specificity, we were also able to show that the therapeutic activity of the same anti-CD7 IT in NOD/SCID mice bearing HSB-2 human leukaemia xenografts is significantly reduced in comparison to that seen in the SCID-HSB-2 model [5]. This latter observation argues for the likely importance of the NK cell as the major cytotoxic effector responsible for ADCC augmentation of IT cytotoxicity in the SCID-HSB-2 model consequent to the established reduced functional activity of NK cells in NOD/SCID mice [6]. These observations also support our working hypothesis that the divergent apoptotic pathways initiated by NK cell delivered granzymes [7] and antibody-mediated delivery of the ribosome inactivating protein (rip) saporin [8,9] act synergistically to achieve a greater level of target cell killing. We hypothesised that stimulating ADCC activity could well increase the therapeutic efficacy of IT treatment further still and the present study was conducted to explore this concept. Poly inosinic:cytidylic acid ([poly (I:C)]), is a TLR3 agonist that mimics double stranded viral RNA [10,11] and is well described as an agent that stimulates NK cell activity [12,13]. In the current study, we show that in vivo activation of NK cells with poly (I:C)] in SCID mice xenografted with human T-ALL cells leads to complete cures of animals treated with the saporin-based anti-CD7 IT HB2-SAPORIN in comparison to only a 50% cure rate in animals treated with HB2-SAPORIN monotherapy. We speculate that this improvement is due to the increased efficiency with which [poly (I:C)] activated NK cells are able to deliver cytotoxic granzymes via ADCC to the target HSB-2 cell cytosol, which then act in concert with the rip saporin to increase target cell killing.

## 2. Experimental Section

### 2.1. Reagents

Poly inosinic: poly cytidylic acid([poly (I:C)]) was obtained from Sigma Aldrich (Poole, UK) (Cat No P9582).

### 2.2. SCID Mice

Pathogen free CB-17 SCID/SCID (severe combined immunodeficient, SCID) mice of both sexes 6–10 weeks of age were produced from our own breeding colony, maintained under British Government Home Office regulations prevalent at the time, and were used in all the experimental work described here. All manipulations on experimental animals were carried out aseptically in a laminar flow hood. Animals were maintained in groups of five as a single sex in filter top microisolator cages and provided with sterile food and water ad libitum. For all experimental work, equal numbers of male and female animals were used for each experimental treatment or control group.

### 2.3. Cell Lines

The CD7^+^ human cell line HSB-2—originally established from peripheral blood leukaemic blasts from a four years old paediatric patient with terminal T-cell acute lymphoblastic leukaemia [14]—was obtained from the European Collection of Cell Cultures (ECACC, Porton Down, Salisbury, UK). HSB-2 cells were authenticated using the Identifier Plus DNA profiling system (Applied Biosciences, Carlsbad, CA, USA). The murine lymphoma cell line, the YAC-1 cell line, which is constitutively sensitive to NK cell-mediated lysis, was also obtained from ECACC and has been described previously [14]. Both cell lines were maintained in the logarithmic phase of growth in culture flasks containing antibiotic-free RPMI 1640 medium with 10% foetal calf serum and supplements of 2 mM sodium pyruvate and 2 mM glutamine (referred to as R10 medium) at 37 °C under a humidified atmosphere of 5% CO_2_.

### 2.4. Antibodies and Antibody F(ab’)_2_ Fragments

The rat anti-mouse antibodies, AT37 (anti-CD2), 6D5 (anti-CD19), and F4/80 (anti-macrophage), and the mouse anti-mouse NK cell antibody, NK1.1 directly conjugated to phycoerythrin, were obtained from Serotec, (Kidlington, UK). The rat antibody 2.4G2 directed against mouse FcγR II/III (anti-CD16/32) was obtained from Pharmingen (San Diego, CA, USA). 

The anti-human CD7 murine antibody, HB2 (clone 3A1), was obtained from the American Tissue Culture Collection (ATCC, Bethesda, MD, USA) and produced in house by hollow fibre fermentation and purified to homogeneity by ion exchange chromatography and gel filtration. F(ab′)_2_ fragments of HB2 antibody were produced by pepsin digestion of native HB2 antibody employing an ImmunoPure F(ab′)_2_ kit (Pierce, Rockford, IL, USA) following the manufacturer’s instructions. F(ab′)_2_ HB2 antibody produced in this way gave a single band of a 110 kD molecular weight on SDS-PAGE analysis under non-reducing conditions and appeared to be wholly free of contaminating Fc.

### 2.5. Immunotoxin Construction

The immunotoxins, HB2-SAPORIN, constructed with intact HB2 antibody and its F(ab′)_2_ equivalent, HB2-F(ab′)_2_-SAPORIN, were constructed by conjugating HB2 antibody or its F(ab′)_2_ fragment to saporin with the heterobifunctional cross linking reagent, *N*-succinimidyl 3-(2-pyridyldithio)propionate (SPDP), as described previously [15]. Only IT containing 2 saporin moieties per IT molecule were used in these studies because of their well-defined characteristics and potency as described by us previously [15]. The purity of the immunotoxins was confirmed by SDS-PAGE and they were then dialysed into PBS pH 7.2, sterilized by passage through a 0.2 μm filter, and stored deep frozen in 100 μg aliquots at −80 °C.

### 2.6. Chromium Release Assay

A chromium release assay was used to assess the constitutive lytic capabilities of SCID mouse splenocytes for HSB-2 and YAC-1 cells in the absence of HB2 antibody or in the presence of increasing amounts of intact or F(ab′)_2_ HB2 antibody in an antibody dependent cellular cytotoxicity (ADCC) assay described previously [2].

### 2.7. Flow Cytometry

Single colour flow cytometry for CD2, FCγRII/III, NK1.1, and F4/80 expression on SCID mouse splenocytes taken at various time points after sham and [poly (I:C)] treatment of SCID mice was carried out on a Beckman Coulter Epics XL flow cytometer and results were analysed using the EPICS software package.

### 2.8. XTT cytotoxicity assay

HSB-2 target cells were exposed to medium alone, 1 or 10 µg/mL [poly(I:C)] for 24 h prior to the assay. The cytotoxicity of HB2-SAPORIN was then determined for triplicate cultures of 5 × 10^4^ target cells receiving each treatment at varying concentrations of IT using an XTT assay with the tetrazolium salt Sodium 2,3,-bis(2-methoxy-4-nitro-5-sulfophenyl)-5-[(phenylamino)-carbonyl]-2H-tetrazolium as previously described by Scudiero et al [16]. The plates were read on a BMG Fluostar Omega plate reader (BMG Labtech, Aylesbury, UK) using a spectral scan from 300 to 650 nm. Results were expressed as a percentage relative to control cells cultured in medium or [poly (I:C)] alone and dose-response curves constructed accordingly.

### 2.9. Cell Proliferation Assay

T25 culture flasks were seeded with 2 × 10^5^ Daudi cells in 10 mL of R10 medium or R10 containing 100 mg/mL [poly(I:C)]. Viable cell counts using trypan blue exclusion were carried out on cultures every 24 h for a 7 days period and the growth curve was determined for each treatment.

### 2.10. Quantitation of IL-2, IL-12, and IFNγ in SCID Mouse Serum

Commercial ELISA-based kits were used to measure the serum levels of murine IL-2, IL-12 (Biosource International Inc, Camarillo, CA, USA Cat #KMC0020-SB & KM0120-SB, respectively), and IFNγ (Cytelisa brand from Cytimmune Sciences Inc, Rockville, MD, USA), adhering to the manufacturer’s instructions.

### 2.11. SCID Mouse Experiments

All animal experiments were carried out in full compliance with British Government Home Office regulations under licence number PIL 70/617 and with local ethics approval in compliance with institutional regulations No PCD 70-2906. On day one of study, groups of 10 SCID mice were injected with two million HSB-2 cells via the tail vein in a 200 μL volume of R10 medium. The growth and dissemination of HSB-2 human T-ALL in SCID mice has been described in detail by us previously [17]. Animals were monitored daily for signs of disease and any animal seen to be in distress was euthanized painlessly and the presence of leukaemia deposits in any internal organ or blood determined by post mortem examination. [poly (I:C)] (10 μg) was administered as a 100 μL bolus i.v. injection in PBS into the tail vein of the appropriate animal groups 24 h before treatment with antibodies or ITs. Appropriate groups were treated with a 6.25 nM amount of intact or F(ab′)_2_ HB2 antibody, HB2-SAPORIN, or HB2-F(ab′)_2_-SAPORIN in a 200 μL volume of PBS administered as a single i.v. bolus injection into the tail vein seven days after HSB-2 cell injection. Groups of 10 control animals were sham treated with 200 μL of PBS with or without [poly (I:C)] as appropriate also on day 7.

### 2.12. Statistical Analyses

#### 2.12.1. Log-Rank Analysis 

Kaplan-Meier Survival Analysis (Holm-Sidak method) was carried out by using the SigmaPlot Software application (Systat Software UK Limited). *p* values of ≤0.05 were considered as statistically significant.

#### 2.12.2. Other Stats Tests

For the flow cytometry experiments to test the level of significance of differences between the experimental groups and the appropriate controls, Microsoft Excel was used to carry out an F-test to test the null hypothesis that the variances of two populations were equal. This was then followed by a two sample T-test, either assuming equal or unequal variance as determined by the F-test. *p* values of ≤0.05 obtained in this way were considered as statistically significant.

## 3. Results

### 3.1. Effects of Timing and Dosage with [poly (I:C)] on ADCC Activity of SCID Mouse Splenocytes

**Figure 1:**
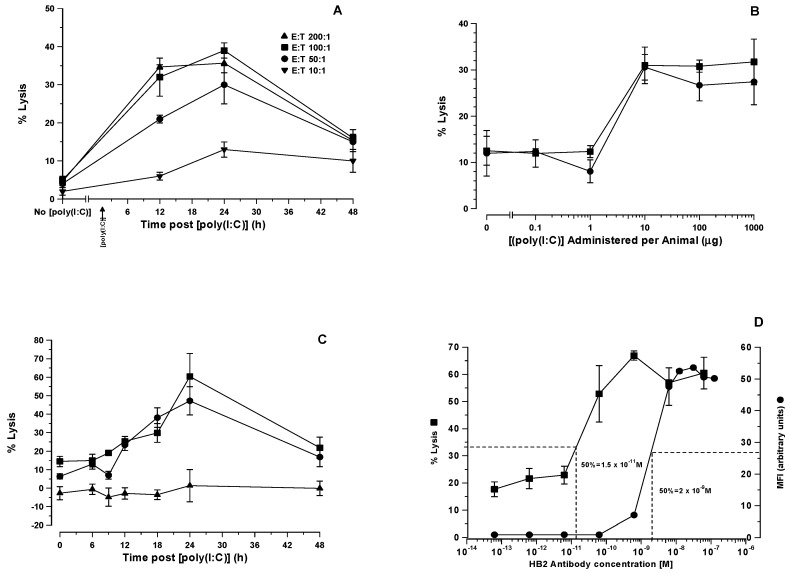
Lytic characteristics of effector splenocytes taken from SCID mice stimulated with [poly (I:C)]. (**A**) Percentage lysis of YAC-1 target cells following incubation with effector splenocytes taken from SCID mice at 12, 24, and 48 h after i.v. injection of 10 μg of [poly (I:C)] at E:T (effector:target cell) ratios of 10:1, 25:1, 50:1, and 100:1 (**B**) Percentage lysis of HB2 antibody coated HSB-2 cells (treated at HB2 antibody concentration of 6.25 × 10^−11^ M ● and 6.25 × 10^−10^ M ■) following incubation with splenocytes from SCID mice injected i.v. 24 h previously with [poly (I:C)] at 0, 0.1, 1, 10, 100, and 1000 μg/animal at an E:T ratio of 100:1 (**C**) Percentage lysis of HSB-2 cells treated with HB2 antibody concentrations of 6.25 × 10^−11^ M ●, 6.25 × 10^−10^ M ■, and an off-target anti-CD19 control antibody, BU12, at 6.25 × 10^−10^ M ▲ following incubation with splenocytes taken from SCID mice at various times following i.v. injection with 100 μg [poly (I:C)]. (**D**) Lytic characteristics of SCID splenocytes at a 1:100 E:T ratio for HSB-2 cells in the presence of various concentrations of HB2 antibody ■ plotted alongside the mean fluorescence intensity (MFI in arbitrary units) seen over the same antibody concentration range.

Firstly, we determined in a ^57^Cr release assay of natural cytotoxicity using YAC-1 as target cells and SCID mouse splenocytes as lytic effector cells, the effector to target (E:T) ratio and the timing of i.v. administration of 100 μg [poly (I:C)] that gave optimal lysis of YAC-1 target cells. The results in Figure 1A show that an E:T ratio of 100:1 is optimal and that maximal lysis occurs at 24 h. We subsequently used an E:T ratio of 100:1 throughout these studies.

Next, we undertook studies on the timing and dosage of [poly (I:C)] administered i.v. to SCID mice in order to optimise ADCC mediated by presumptive activated NK cells within the splenocyte population against HB2 antibody coated HSB-2 target cells. In the first instance, we administered various doses of [poly (I:C)] i.v. (range 0.1 to 1000 μg/animal) to groups of three SCID mice and measured the ability of pooled splenocytes taken from these animals at 24 h after administration to lysed HSB-2 cells exposed continuously to two different concentrations of HB2 antibody. At a [poly (I:C)] dose of 1 μg, there was no observed increase in lytic activity above the baseline, but at a dose of 10 μg, there was a tripling from the 12% baseline level to 31% (Figure 1B). This was seen with both concentrations of HB2 antibody employed. Furthermore, there were no further increases in lytic activity when splenocytes were taken from animals treated at higher [poly (I:C)] dose levels.

In a further experiment, we took splenocytes from SCID mice that had been injected i.v. with 10 μg [poly (I:C)] at various times ranging from 6 to 48 h after administration and tested their ability to lyse HSB-2 cells in the presence of two concentrations of intact anti-CD7 HB2 antibody or the off-target intact anti-CD19 antibody, BU12, used at the highest concentration of 6.25 × 10^−10^ M. The lysis of HSB-2 cells increased with time in the presence of the HB2 antibody, with little difference between the two HB2 antibody concentrations, both peaking at 60% lysis by 24 h and declining thereafter to 21% lysis by 48 h as shown in Figure 1C. No lysis of HSB-2 cells occurred with the off-target control BU12 antibody.

### 3.2. Maximal ADCC Against HSB-2 Target Cells Occurs at Sub-Saturating Concentrations of HB2 Antibody

To determine the extent of CD7 receptor saturation by HB2 antibody that is required to maximally lyse HSB-2 target cells, we conducted a study where the mean fluorescent intensity (MFI) produced by different concentrations of antibody was compared with the extent of lysis produced by the same concentration of antibody in an ADCC assay using [poly (I:C)] activated SCID mouse splenocytes as cytotoxic effectors. The results are shown in Figure 1D. Maximal lysis of HSB-2 cells (67%) occurred when CD7 receptors on the cell surface were only 13% saturated and plateaued thereafter when higher antibody concentrations were used. Even when <1% of cell bound antibody was detected by flow cytometry at an antibody concentration of 6.25 × 10^−10^ M, 53% lysis of HSB-2 cells was still observed. Below this threshold antibody concentration, however, lysis occurred only at baseline levels (Figure 1D).

### 3.3. Kinetics of Changes in Serum IL-2, IL-12, and IFNγ Levels in SCID Mice Treated with [poly (I:C)]

**Figure 2:**
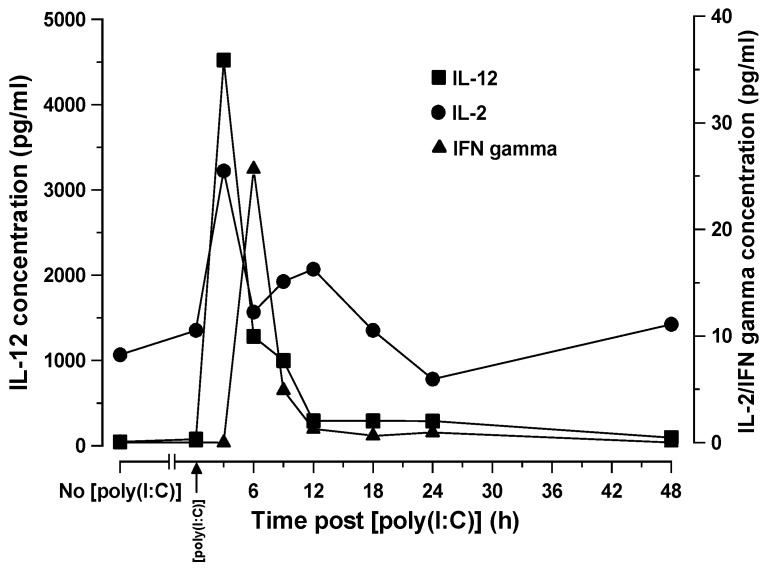
Serum concentrations of IL-2, IL-12, and INFγ at various time points out to 48 h after i.v. injection of 100 μg of [poly (I:C)] into SCID mice.

The time related changes in serum levels of IL-2, IL12, and IFNγ in SCID mice treated i.v. with 10 μg [poly (I:C)] were measured and are shown in Figure 2. IL-2 and IL-12 levels both peaked three hours after administration, achieving levels of 25 pg/mL and 4550 pg/mL, respectively. The IL-12 level showed a simple rapid decline and returned to pre-treatment levels by 12 h. In contrast, the decline in IL-2 levels appeared to show a biphasic pattern, with a rapid initial decline up to 6 h, followed by a modest increase once again up to 9 h, followed by a slower decline once again until 24 h, whereupon pre-treatment levels were reached. The IFNγ level peaked by 6 h at 25 pg/mL and was therefore 6 h later than the IL-12 and IL-2 peak level times. IFNγ levels rapidly returned to the near pre-treatment level by 12 h.

### 3.4. Expression Kinetics of FcγRII/III, NK1.1, and F4/80 by SCID Mouse Splenocytes Following In Vivo Treatment with [poly (I:C)].

**Figure 3:**
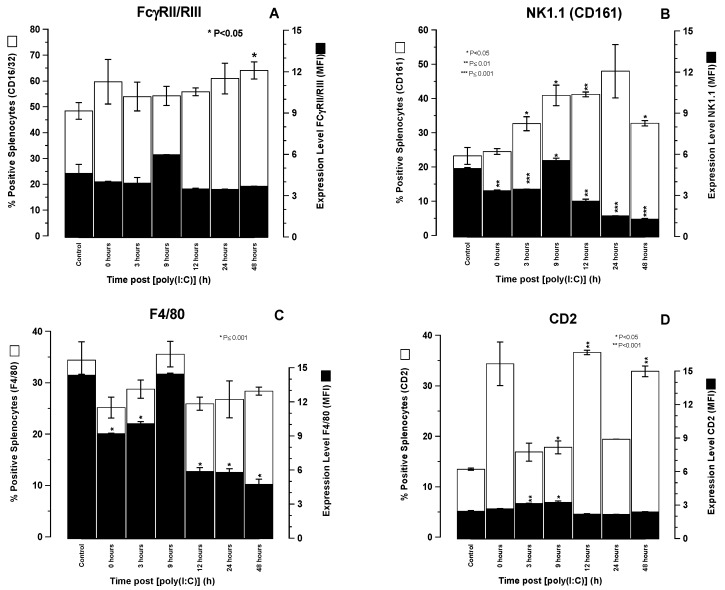
Percentage of SCID mouse splenocytes positive (□) for cell surface FCγRII/RIII (**A**), NK1.1 (CD161) (**B**), F4/80 (**C**), and CD2 (**D**) and their respective expression levels (■) shown as mean fluorescent intensity (MFI) at various time points following i.v. injection of 100 mg [poly (I:C)]. Error bars show standard deviations obtained from three replicate samples. Asterisks shown within individual chartlets denote the level of significant differences between read outs obtained from untreated control splenocytes and splenocytes from animals at various time points after i.v. treatment with 100 μg [poly (I:C)].

Figure 3A–D show the expression levels and proportion of cells within the global SCID mouse splenocyte population expressing (A) FcγRII/III (CD16/32), (B) NK1.1 (CD161), (C) F4/80 (an unclustered mouse macrophage marker), and (D) CD2 at various time points up to 48 hours after i.v. administration of 10 μg [poly (I:C)]. Each time point represents results obtained for pooled splenocytes from three individual SCID mice measured in triplicate by single colour flow cytometry.

The number of splenocytes expressing FcγRII/III remained fairly constant throughout the observation period, with the exception of a small though significant (*p* < 0.05) increase observed at 48 h. The expression levels (MFI) of FcγRII/III also remained fairly constant throughout the observation period, with a modest though non-significant increase at 9 h (Figure 3A).

In contrast the percentage of splenic cells expressing NK1.1 (CD161) had increased significantly by 3 h (*p* < 0.05), almost doubling in number from 23% to 41% by 12 h (*p* < 0.01) then declining to 31% by 48 h (Figure 3B). The increase in the number of NK1.1^+^ splenic cells was associated with concomitant significant decreases in the expression level of NK1.1 that reached a nadir 48 h after [poly (I:C)] treatment (Figure 3B). 

Over the same time course, there were also fluctuations in the absolute numbers of splenocytes expressing the unclustered macrophage marker, F4/80, but none of these were significantly different to the untreated controls. However, the expression level of F4/80 fell more than half compared to controls at 12, 24, and 48 h with a significance level of *p* < 0.001. (Figure 3C). 

During the same 48 h time course, there were only apparently minor changes in the expression levels of CD2 within the splenic population and although small these were significant (*p* < 0.05) at the 3 and 6 h time points (Figure 3D). There were obvious significant peaks at 12 and 48 h in the numbers of CD2 expressing cells after [poly (I:C)] administration (*p* < 0.001). The peak cell number at 0 h was not significant. 

### 3.5. Lysis of HB2 Ab or HB2-Saporin IT Coated HSB-2 Target Cells by Splenocytes from SCID Mice Treated with [poly (I:C)]

**Figure 4:**
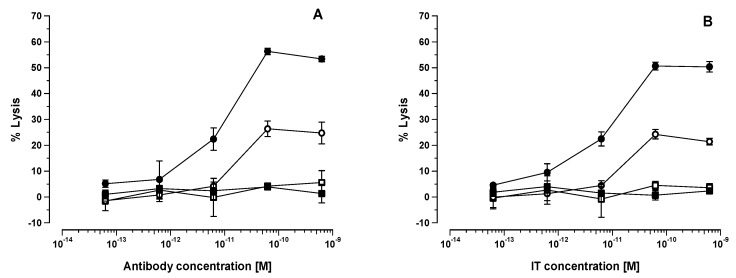
Lysis of target HSB-2 cells in a ^56^Cr release assay by splenocytes at a 1:100 T:E ratio from untreated SCID mice (open symbols) or following treatment 24 h previously with 100 μg [poly (I:C)] i.v. (closed symbols) in the presence of varying concentrations of (**A**) intact HB2 antibody (⚬, ●), or Fab_2_ HB2 antibody (□, ■) or (**B**) HB2-SAPORIN IT (⚬, ●), HB2-F(ab’)_2_-SAPORIN (□, ■).

Results obtained for the immunospecific lysis of HSB-2 cells coated with various concentrations of HB2 antibody, F(ab’)_2_-HB2 antibody, HB2-SAPORIN IT, or F(ab’)_2_-HB2-SAPORIN IT in an ADCC assay using SCID mouse splenocytes from untreated animals or 24 h after i.v. injection of 10 μg [poly (I:C)] are shown in Figure 4A,B. HB2 antibody and HB2-SAPORIN IT gave virtually identical lysis curves when used against HSB-2 target cells that was markedly increased for both when splenocytes activated in vivo by [poly (I:C)] were used as the cytotoxic effector cells. The F(ab’)_2_ derivatives for HB2 Ab and IT were both incapable of eliciting lysis with either untreated or [poly (I:C)] treated splenocytes as effectors, indicating the absolute requirement of the Fc domain for lysis.

### 3.6. The Effects of [poly (I:C)] on CD7 Expression, Proliferation, and Sensitivity of HSB-2 Cells to HB2-SAPORIN

**Figure 5:**
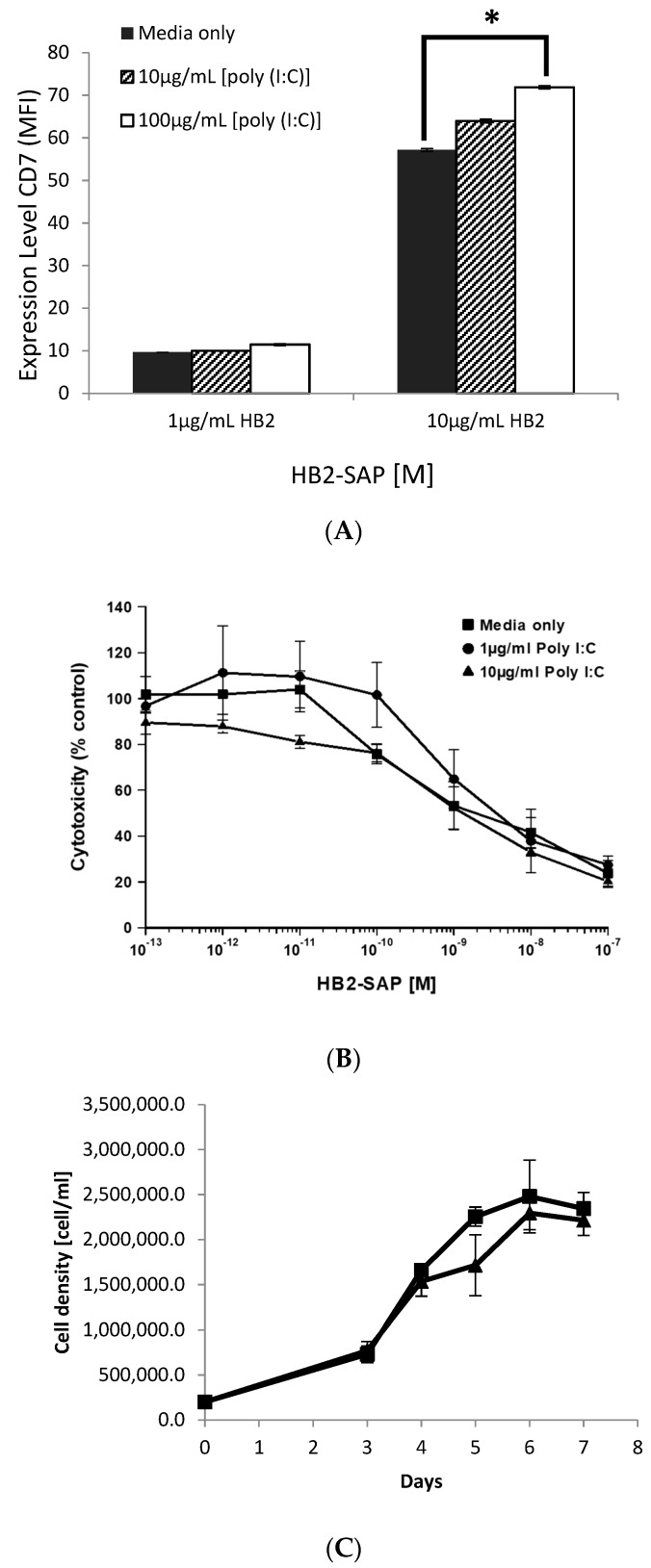
The in vitro effects of [poly (I:C)] on HSB-2 cells. (**A**) Expression levels of CD7 determined by single colour flow cytometry with HB2-SAPORIN IT at 1 or 10 µg/mL following 24 h exposure to medium only (controls) or [poly (I:C)] at 10 or 100 μg/mL for 24 h. * *p* < 0.01 as determined by two sample T-test. (**B**) Cytotoxicity of HB2-SAP IT measured by XTT assay for control HSB-2 cells (■) or cells exposed to 1 (●) or 10 μg/mL (▲) [poly (I:C)] for 24 h prior to assay (**C**) Outgrowth of HSB-2 control cells (■) or cells continuously exposed to 100 mg/mL [poly (I:C)] (▲) over a six day period.

To exclude the possibility that [poly (I:C)] was exerting a direct effect on HSB-2 cells by changing their expression level of CD7, rendering them more sensitive to the cytotoxic effects of the HB2-SAPORIN IT, or through a direct cytotoxic effect, we undertook three separate experiments. Firstly, we treated HSB-2 cells in culture with 10 or 100 µg/mL [poly (I:C)] for 24 h and then measured the expression levels of CD7 by flow cytometry. [poly (I:C)] treatment at 10 µg/mL had no significant effect on CD7 expression levels, but at 100 µg/mL, it resulted in a significant (*p* < 0.01) increase in expression from 57 to 72 MFI arbitrary units (Figure 5A). Secondly, we measured the proliferation rate of HSB-2 cells cultured in standard R10 media or in R10 medium containing 10 µg/mL [poly (I:C)] and observed that [poly (I:C)] had only a minor inhibitory effect on the in vitro proliferation rate (Figure 5B). Thirdly, we investigated whether [poly (I:C)] affected the sensitivity of HSB-2 cells to HB2-SAPORIN IT by comparing the dose-response curves obtained in an XTT assay conducted in the absence and presence of 1 or 10 µg/mL [poly (I:C)]. We found that [poly (I:C)] had no significant observable effect on the cytotoxicity of HB2-SAPORIN for HSB-2 cells (Figure 5C).

### 3.7. Effects of [poly (I:C)] on Therapy Outcomes with HB2 Antibody or HB2-SAPORIN IT in SCID-HSB-2 Mice

**Figure 6:**
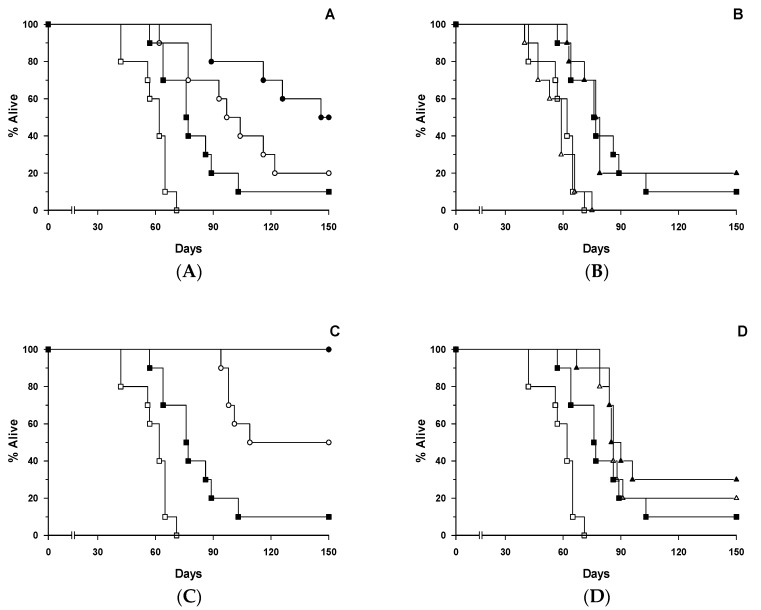
Kaplan-Meier survival curves for SCID-HSB-2 mice receiving a single bolus i.v. injection of phosphate buffered saline (PBS) (open symbols) or a single i.v. bolus injection of [poly(I:C)] (closed symbols) 24 h in advance of treatment with (**A**) intact HB2 antibody (◯, ⬤) or PBS (☐, ⬛), (**B**) a F(ab’)_2_ fragment of HB2 antibody (△, ▲) or PBS (☐, ⬛), (**C**) HB2-SAP IT (◯, ⬤) or PBS (☐, ⬛), and (**D**) HB2-F(ab)_2−_SAP (△, ▲) or PBS (☐, ⬛). Log-rank analysis was used to evaluate significant differences between treatment groups and *p* values are reported in 3.7 under the results section.

Kaplan-Meier survival curves showing the therapeutic effect of HB2 intact and F(ab’)_2_ antibody fragments or HB2-SAPORIN ITs constructed with the equivalent intact antibody or F(ab’)_2_ fragment in SCID-HSB-2 mice that had been injected 24 h prior to antibody therapy with 100 µg/mL [poly (I:C)] or sham treated with PBS are shown in Figure 6A–D. [Poly (I:C)] monotherapy did exert a significant therapeutic effect (*p* = 0.00726) compared with PBS sham treated controls though it was not curative, with 90% of animals dying from disseminated leukaemia by day 150 (Figure 6A). HB2 antibody monotherapy also exerted a significant (*p* < 0.05) therapeutic effect in non-[poly (I:C)] treated animals, but the therapeutic effect of intact HB2 antibody was further significantly improved (*p* = 0.04) when animals were treated 24 h previously with [poly (I:C)], resulting in 50% disease-free survival (dfs) at 150 days compared with 20% dfs in the antibody monotherapy treated group (Figure 6A). The HB2-F(ab’)_2_ antibody fragment had no therapeutic effect in SCID-HSB-2 mice used either with or without [poly (I:C)] when compared with [poly (I:C)] treated controls (Figure 6B). Survival curves for groups of SCID-HSB-2 mice treated with HB2-SAPORIN IT with and without [poly (I:C)] are shown in Figure 6C. HB2-SAPORIN IT exerted a significant therapeutic effect compared to PBS or [poly (I:C)] treated controls (*p* = 0.0000312 and *p* = 0.0075, respectively) with 50% dfs at 150 days. Treatment of SCID-HSB-2 mice with [poly (I:C)] 24 h prior to IT resulted in a 100% dfs (Figure 6C). HB2-F(ab’)2-SAPORIN IT had a significantly reduced therapeutic effect compared with HB2-SAPORIN constructed with intact antibody (Figure 6C,D). Prior administration of [poly (I:C)] did not significantly influence the therapeutic effect of the HB2-F(ab’)_2_-SAPORIN IT (Figure 6D), which gave a survival curve very similar to the [Poly (I:C)] monotherapy treated group.

## 4. Discussion

The main finding to emerge from this study is that [poly (I:C)] administered i.v. to SCID mice xenografted with a disseminated human T-ALL cell line significantly increased the therapeutic effectiveness of the anti-CD7 HB2-SAPORIN IT, leading to a 100% cure rate in the [poly(I:C)]/IT combination treated animals. This is in comparison with only a 50% cure rate when IT was used without prior [poly (I:C)] treatment. Similarly, [poly (I:C)] significantly increased the therapeutic activity of naked HB2 antibody in this SCID model. Utilising the same SCID-HSB-2 xenograft model, we have previously demonstrated that host-mediated ADCC significantly contributes to the therapeutic effect of the same anti-CD7 IT, HB2-SAPORIN. In the present study, we showed that in vivo activation of putative SCID mouse NK1.1^+^ effector cells with the TLR3 agonist [poly (I:C)] provides an additional boost to augmentation and a resultant further significant improvement to the therapeutic effect of an anti-CD7-saporin IT in the SCID-HSB-2 model. [Poly (I:C)] has been shown to have direct apoptotic and growth inhibitory effects on various human cancer cell lines [18,19], and, furthermore, is capable of augmenting the anti-tumour effects of ErbB2 antibodies for breast cancer cell lines through a putative ADCC mechanism [20]. We were able discount these possibilities by conducting various experiments that excluded a direct cytotoxic or growth inhibitory effect of [poly (I:C)] on target HSB-2 cells. Our studies also excluded any direct augmentative effect of [poly (I:C)] on IT cytotoxicity demonstrable using the XTT assay. We therefore conclude that [poly (I:C)] likely exerts an augmentative effect through an in vivo immunological mechanism(s) probably via the stimulation of ADCC driven by [poly (I:C)] activated NK cells and maybe other myeloid derived cytotoxic effector cells found in SCID mice also activated by [poly (I:C)] [21,22].

Early studies with [poly (I:C)] showed that this agent exerted anti-viral and anti-tumour effects that were ascribed to its ability to stimulate IL-12 production with subsequent NK cell activation that resulted in γ-interferon release by these cytotoxic effectors [12]. In this context, Guinn et al. showed that [poly (I:C)] synergized with IFNγ to inhibit murine and human cancer cell growth by influencing cell cycle control via cyclin D1 and the induction of the intrinsic apoptosis pathway [23]. The extrinsic cellular dsRNA sensing pathways for TLR3 agonists, such as [poly (I:C)], and related molecules have now been relatively well defined in terms of the intracellular signalling cascades and cytokine/chemokine outputs [24]. In this context, previous studies have shown that [poly (I:C)] treatment of macrophages induces downstream activation of NFκB and MAP kinases, leading to transcription of key cytokines that drive an innate immune response [10,25]. In the mouse, TLR3 is expressed by plasmacytoid dendritic cells, myeloid dendritic cells, monocytes, and macrophages mainly confined to the endoplasmic reticulum, but also, in some cases, on the plasma membrane surface [26]. Later work showed that [poly (I:C)] was selectively recognised and bound by TLR3 expressed by murine macrophages, which then resulted in the downstream activation of NFκB [10,27]. Also of direct relevance to our study, [poly (I:C)] has been shown to significantly increase the therapeutic activity of an anti-ErbB2 antibody in a mouse model of breast cancer, an outcome that was interpreted by these workers as being due to an increase in the activity of NK and CD8^+^ cytotoxic T-cells [20]. More recently, using a BCL_1_ murine lymphoma model, Dahal et al. [28] were able to show that the lymphoma created a tumour suppressive microenvironment that had an adverse effect on anti-CD20 antibody directed treatment that was due, at least partially, to an increase in the expression level of inhibitory FcγRIIB on tumour associated macrophages (TAM). [Poly (I:C)] reversed this effect in vitro, shifting the FcγRIIA:FCγRIIB (stimulatory:inhibitory) ratio in favour of a TAM phenotype capable of appropriately engaging antibody Fc to elicit ADCC. [Poly (I:C)] also partially reversed this effect in an adoptive transfer in vivo model. [Poly (I:C)] has also been shown to exert an effect on myeloid derived suppressor cells (MDSC) within the tumour microenvironment of a murine model of breast cancer, reducing the immunosuppressive properties of MDSC [29]. Such a mechanism is, however, unlikely to contribute to the therapeutic effect of [poly (I:C)] described here in our SCID-HSB-2 model due to a lack of functional CD8^+^ or CD4^+^ cytotoxic effectors in SCID mice. Possibly more relevant to our own findings, Shime et al. [21] demonstrated in a Lewis lung cancer mouse model that [poly (I:C)] polarised TAMs from a tumour supporting phenotype to an M1-type tumour suppressor phenotype, which then exerted a significant anti-tumour effect. Because SCID mice do possess functional macrophages [6], a similar mechanism may well be operative in the SCID-HSB-2 model described here. Additional studies are required to determine this. 

In the current study, the time related cytokine profiles we observed in SCID mice after [poly (I:C)] administration are consistent with a multi-tiered activation process as previously described [25]. This indicates that the cellular populations responsible for activating NK cells, which subsequently elicit ADCC, are intact in the SCID mouse and are therefore unaffected by the functional deficiency in SCID T- and B-cell populations. SCID mice, however, do possess fully functional monocytes, macrophages, dendritic cells, NK cells, and the full complement of myeloid cell populations [6] that might also participate as cytotoxic effector cells and cannot, therefore, be excluded as being contributory. 

The present study has, therefore, not confirmed that the NK1.1^+^ cell is the sole cytotoxic effector in this model system. However, experimental evidence from our previous studies using anti-asialo GM1 poly immunoglobulins (pIg) antibody to deplete NK cells in vivo taken together with our observations in NOD/SCID mice deficient in functional NK cells (but possessing macrophages and other myeloid-derived cells) suggests that the NK cell is likely to be the major, but not necessarily the sole, participating cytotoxic effector cell [2]. As described by us previously and from the in vitro and in vivo experiments described in the present study, we have shown unequivocally the absolute necessity of an Fc domain to elicit the augmentative effect, indicating that the engagement of Fc with putative FcγRIII on the NK cell surface and the subsequent formation of an immunological synapse (NKIS) between the NK cell and target cell is a prerequisite to achieve an augmentative effect. As predicted, the lack of an Fc domain in the HB2-F(ab’)_2_-SAP IT abolished its in vitro ADCC activity for target HSB-2 cells and furthermore also significantly diminished the in vivo therapeutic effect of this IT in SCID-HSB-2 mice. It is important to note that [poly (I:C)] monotherapy did exert a significant, though non-curative, anti-leukaemia effect in this SCID-HSB-2 model consistent with observations made by others in a variety of tumour systems [30]. Our experimental results indicate that this is not due to the direct action of [poly (I:C)] on the HSB-2 cell, but is rather likely mediated through the activation of cytotoxic NK cells or other cytotoxic effector cell population(s) in the host. It is also important to note that the therapeutic effect of HB2-F(ab’)_2_-SAP IT was significantly diminished in comparison to the HB2-SAPORIN IT constructed with intact HB2 antibody. When HB2-F(ab’)_2_-SAP IT was used in SCID-HSB-2 mice that had been pre-treated 24 h previously with [poly (I:C)], there was no improvement in the therapeutic effect above that was seen in [poly (I:C)] monotherapy treated animals. To the contrary, we observed that HB2-SAPORIN containing an intact Fc domain exerted a significantly improved therapeutic effect (*p* < 0.01) with a 100% cure rate in SCID-HSB-2 mice pre-treated with [poly (I:C)] when compared with to SCID-HSB-2 mice that were not pre-treated with [poly (I:C)]. This clearly indicates the importance of the Fc domain to achieve an augmentative effect not only in standard SCID-HSB-2 mice, but also in animals pre-treated with [poly (I:C)]. We interpret this to mean that [poly (I:C)] treated SCID-HSB-2 mice possess activated NK cells that participate more effectively in ADCC, which in turn co-operates with and reinforces IT-mediated killing of CD7^+^ HSB-2 cells.

We have demonstrated in the present study that maximal lysis of HSB-2 cells (67%) occurs when only 13% of CD7 receptors on the cell surface were occupied by antibody, plateauing thereafter at higher antibody concentrations. There is an average of 72,500 CD7 molecules expressed on the surface of the sub-clone of HSB-2 cells used in this study [31]. Thirteen percent occupancy of CD7 receptors by the antibody therefore equates to approximately 9418 CD7 molecules sufficient to achieve maximal lysis whilst occupancy of as few as 724 sites (1% saturation) still delivered 53% lysis. The absolute threshold below which lysis does not occur therefore lies somewhere between 724 and 72 CD7 sites bound by cognate HB2 antibody, a remarkably low figure that attests to the efficiency of ADCC in this particular model system. 

We propose a mechanistic model to explain this, which speculates that perforin-dependent delivery of granzymes to target HSB-2 cells by cytotoxic effector cells during ADCC synergise with the ribotoxic effect of IT-mediated saporin-mediated protein synthesis inhibition. GrzA and GzB delivered by NK-cell-mediated killing invoke apoptotic pathways that are radically divergent from each other [7], which in turn are also different from the saporin-mediated apoptotic pathway(s) [8,9]. It is easy to envisage that these different pathways converge on different and/or common death substrates to achieve a more efficient target cell killing. 

Whilst SCID mice are grossly deficient in B- and T-cells and therefore adaptive immunity, their innate immunity is largely intact through their possession of relatively normal numbers of functional NK cells, macrophages, and various cells of myeloid lineage that includes plasmacytoid dendritic cells (PDC) [32,33]. In previous studies, we have shown that 32% and 37% of splenocytes from SCID mice are NK1.1^+^ and FcγRII/III positive, respectively, and that 96% of SCID NK1.1^+^ cells also express FcγRII/III. Their splenocytes can participate in both natural and antibody-dependent cellular cytotoxicity. SCID mouse splenocytes are lytically fully functional in both NC and ACCC, making these the most likely effector cell population responsible for ADCC described in the present study. 

The time-related shifts in serum cytokine levels and immunophenotypically relevant splenocyte populations observed in SCID mice following [poly (I:C)] administration were consistent with what would be expected in immunologically intact mice. Thus, the recognition of [poly (I:C)] as a synthetic viral dsRNA PAMP by cytoplasmic TLR3 within the cytosol of PDC’s resulted in IL-2 and IL-12 production by these host cells that preceded INFγ release from NK cell effectors at 3 h. This two stage activation of NK1.1 cells forms the basis of NK cell activation by PAMPs, such as [poly (I:C)]. The time-related immunophenotypic shifts in splenic cell populations following [poly (I:C)] administration were revealing and showed an increase in the absolute numbers of NK1.1^+^ cells commencing 3 h after [poly (I:C)] administration, peaking at 24 h, and declining thereafter. This increase in NK1.1^+^ cell numbers was accompanied by a general decrease in NK1.1 expression levels possibly indicative of a proliferating cell population in which cell division results in a dilution of the NK1.1 surface antigen density distribution. There were no significant changes in the percentage of splenocytes positive for FcγRII/III other than a small increase at 9 h that was not significant. These data indicate that an increase in NK1.1^+^ cell numbers may account, at least in part, for the increase in ADCC activity, though this cannot be attributed to any overall increase in FcγRII/III receptors to engage antibody Fc. We speculate that it is more likely the increase in the activation status of NK and possibly other cytotoxic effector cells rather than an increase in their numbers that contributes the increased killing efficiency.

The SCID-HSB-2 therapy studies we have described here clearly demonstrate that [poly (I:C)] activation of putative NK cells in the host leads to significant improvements in the therapeutic effect of both HB2 antibody and HB2-SAPORIN IT in comparison to results obtained in non-[poly (I:C)] treated mice. Our data provides a sound rationale in favour of exploring the clinical benefits of such a combinatorial approach and the availability and recent approval of clinical grade [poly (I:C)] (Ampligen) [13] makes this an entirely feasible proposition.
